# Association between household solid fuel use and tuberculosis: cross-sectional data from the Mongolian National Tuberculosis Prevalence Survey

**DOI:** 10.1186/s12199-021-00996-4

**Published:** 2021-08-09

**Authors:** Munkhjargal Dorjravdan, Katsuyasu Kouda, Tsolmon Boldoo, Naranzul Dambaa, Tugsdelger Sovd, Chikako Nakama, Toshimasa Nishiyama

**Affiliations:** 1 Department of Hygiene and Public Health, Kansai Medical University, 2-5-1 Shin-machi, Hirakata, Osaka, 573-1010 Japan; 2 Tuberculosis Surveillance and Research Department, National Center for Communicable Disease, Nam Yan Ju Street, Bayanzurkh district, Ulaanbaatar, 13701 Mongolia; 3 Swiss Tropical and Public Health Institute, Socinstrasse 57, CH-4051 Basel, Switzerland

**Keywords:** Tuberculosis, Household air pollution, Solid fuel, Indoor air pollution, Epidemiology

## Abstract

**Background:**

Tuberculosis (TB) and indoor air pollution (IAP) are equally critical public health issues in the developing world. Mongolia is experiencing the double burden of TB and IAP due to solid fuel combustion. However, no study has assessed the relationship between household solid fuel use and TB in Mongolia. The present study aimed to assess the association between household solid fuel use and TB based on data from the Mongolian National Tuberculosis Prevalence Survey (MNTP Survey).

**Method:**

The MNTP Survey was a nationally representative population-based cross-sectional survey targeting households in Mongolia from 2014 to 2015, with the aim of evaluating the prevalence of TB. The survey adopted a multistage cluster sampling design in accordance with the World Health Organization prevalence survey guidelines. Clusters with at least 500 residents were selected by random sampling. A sample size of 98 clusters with 54,100 participants was estimated to be required for the survey, and 41,450 participants were included in the final analysis of the present study. A structured questionnaire was used to collect information on environmental and individual factors related to TB. Physical examination, chest X-ray, and sputum examinations were also performed to diagnose TB.

**Results:**

The use of solid fuels for heating (adjusted odds ratio (aOR): 1.5; 95% confidence interval (CI): 1.1–2.1), male gender (aOR: 2.2; 95% CI: 1.6–3.2), divorced or widowed (aOR: 2.6; 95% CI: 1.7–3.8), daily smoker (aOR: 1.8; 95% CI: 1.3–2.5), contact with an active TB case (aOR: 1.7; 95% CI: 1.2–2.3), being underweight (aOR: 3.7; 95% CI: 2.4–5.7), and previous history of TB (aOR: 4.3; 95% CI: 3.0–6.1) were significantly associated with bacteriologically confirmed TB after adjusting for confounding variables.

**Conclusion:**

The use of solid fuels for heating was significantly associated with active TB in Mongolian adults. Increased public awareness is needed on the use of household solid fuels, a source of IAP.

## Background

Tuberculosis (TB) is a major public health issue and is one of the leading 10 global causes of death, particularly in low and middle-income countries [1]. According to the World Health Organization (WHO), a majority of the 10 million incident TB cases and 1.4 million TB deaths occurred in low and middle-income countries in 2019 [2]. Mongolia is one of the high TB burden countries in the Western Pacific region, with a TB prevalence of 428 per 100,000 population [3]. Five to 15% of individuals with mycobacterium TB infection develop active TB, particularly when they have risk factors such as an immunosuppressive condition (HIV, diabetes, age, malnutrition), deteriorating socioeconomic conditions, environmental exposure, and behavioral factors (smoking and alcohol consumption) [4]. Indoor air pollution (IAP) from the use of solid fuels (e.g., coal) is a potential risk factor for TB, given the negative impact it has on the airway defense mechanism [5]. A majority of health-related exposure to air pollution from solid fuels occurs around the household in low and middle-income countries [6].

Over 3 billion people continue to rely on household solid fuels (e.g., wood, coal, crop residue, animal dung, and charcoal) and use simple stoves for cooking and heating [7]. According to the 2010 household census in Mongolia, 45.2% of households lived in traditional ghers, 29.5% lived in houses equipped with simple stoves, and 72% used solid fuels in everyday cooking and/or heating [8]. In developing countries, household combustion of solid fuels emits health-damaging pollutants, causing a high level of IAP [9]. Burning solid fuels with inefficient stoves or open hearths produces various pollutants, including particulate matter (PM), methane, carbon monoxide, polyaromatic hydrocarbons, and volatile organic compounds [10, 11]. Exposure to such compounds indoors is likely to have a greater impact on health than exposure to the same compounds outdoors. In fact, around 4.3 million people die globally due to household IAP every year [10]. During the cold season in Mongolia, households burn over 600,000 tons of coal for domestic heating, and 80% of air pollution in the city is caused by household solid fuel combustion [12]. Air pollution is attributed to 9.7% of all deaths in Ulaanbaatar (UB) City, Mongolia’s capital [13].

Many studies have reported a direct relationship between the exposure to household solid fuels and negative health consequences, including chronic obstructive pulmonary disease, acute lower respiratory infection, lung cancer, cardiovascular disease, and cataracts [11, 14, 15]. However, the relationship between household IAP from solid fuel use and active TB remains controversial. A case-control study in India reported a significant positive association between biomass fuel use and pulmonary TB [16]. Yet, other studies did not find strong evidence for a positive association between household solid fuel use and TB [17, 18]. While a systematic review in 2013 reported strong evidence for an association between IAP and TB [19], a more recent systematic review of IAP and TB concluded the level of association between the two to be very low [18]. Another systematic review of solid fuel use and active TB concluded that the risk of active TB is dependent on the type of fuel used, with the highest risk being associated with biomass burning [20]. Regarding other types of fuel, the number of published studies on this topic was small, and the results in some studies did not account for confounding factors [20].

TB is one of the leading causes of mortality from respiratory disease in Mongolia [21], and IAP from solid fuel use is also a major issue [13, 22]. However, there is no evidence supporting a relationship between solid fuel use and active TB in the country. Against this backdrop, the present study aimed to assess the association between household solid fuel use and active TB based on data from the Mongolian National Tuberculosis Prevalence Survey (MNTP Survey), a large-scale study of a representative Mongolian adult population.

## Methods

### Country

Mongolia is divided administratively into 21 provinces and the capital, UB City. Almost half of the population lives in UB City, with 250 people per square kilometer, while provinces have about 2 people per square kilometer [8]. Each province has a rural area and a provincial center. The rural area consists of small rural administrative units called “soum,” and the provincial center is further divided into sub-soums. UB City has 9 districts and 134 sub-districts.

### Sample size estimation

**Fig. 1:**
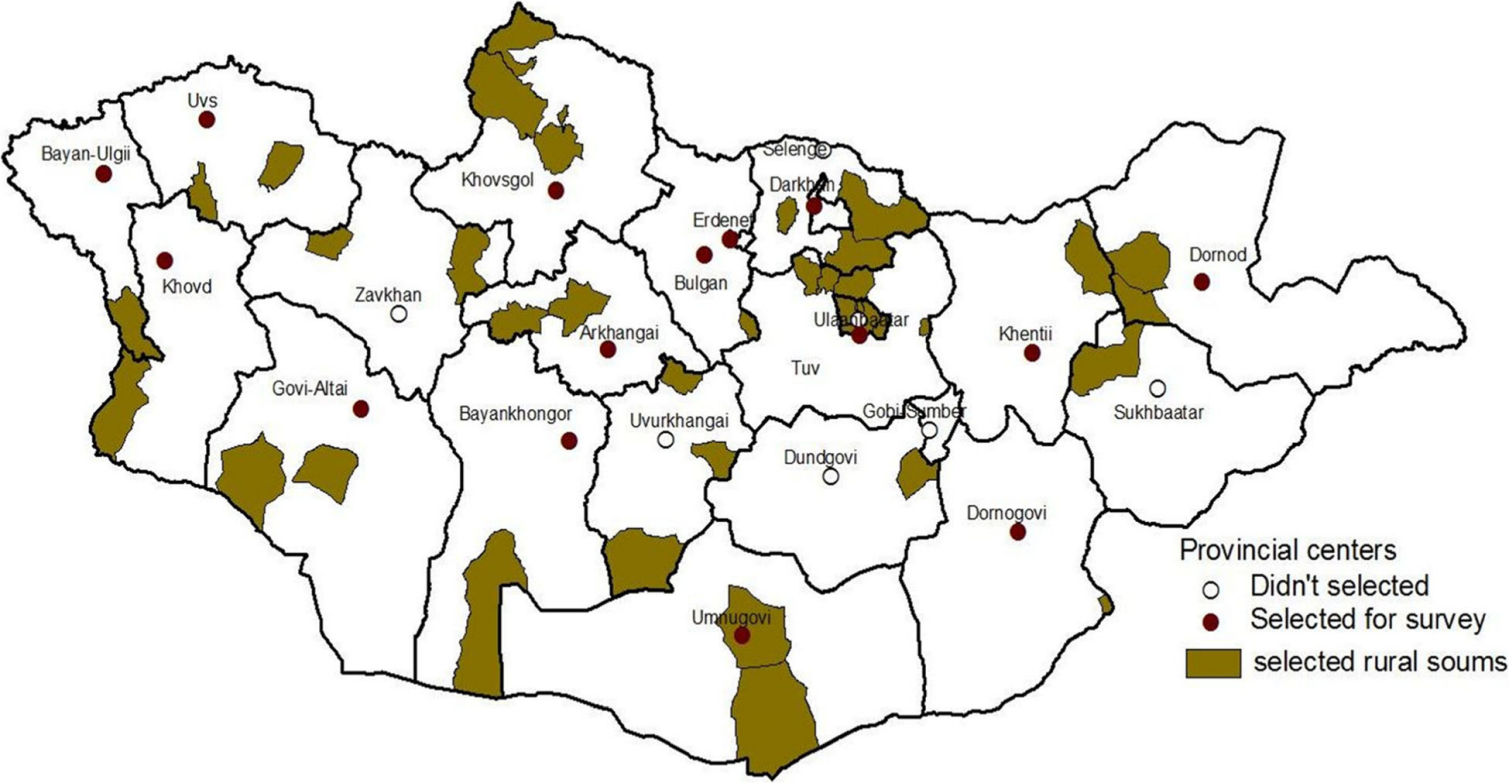
Selected rural soums and provincial centers

The MNTP Survey was a nationally representative population-based cross-sectional survey of households in selected clusters that aimed to investigate the prevalence of TB in Mongolia, and was conducted from April 2014 to November 2015. The 2010 Report of the Population and Housing Census conducted by the Mongolian government was used to define the sampling frame of the MNTP survey [8]. The sample size of the MNTP Survey was calculated using the WHO TB prevalence survey guidelines [23]. The survey population was divided into 3 strata according to settlement type (rural soums, provincial centers, and cities) in order to estimate the prevalence of TB. Primary sampling units (PSUs) in each stratum were defined as a soum in a rural area, a sub-soum in a provincial center, and sub-districts in UB City (Fig. 1). In the first stage, 98 PSUs were recruited from a list of units across Mongolia (36 from rural soums, 15 from provincial centers, and 51 from cities) using the multi-stage, random cluster sampling method. Each PSU consists of several small blocks called “clusters” consisting of ≥ 500 people aged ≥ 15 years. Proportional probability to size sampling was used for primary sampling units, and random sampling was used for cluster sampling from a list of clusters. The required sample size for the survey was 54,100 adults from 98 clusters.

### Analyzed population

**Fig. 2:**
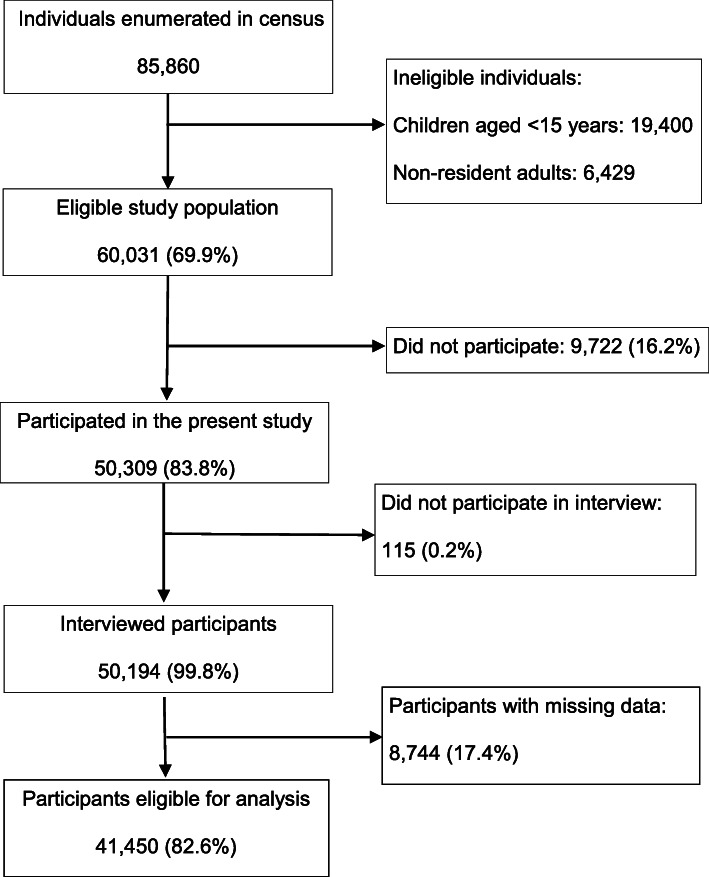
Flowchart of participant selection

After random cluster sampling, a total of 85,860 individuals of all ages in 98 clusters were enumerated (Fig. 2). Among the enumerated individuals, children aged < 15 years (*n* = 19,400) and individuals who did not meet the residential duration criteria (*n* = 6,429) were excluded from the MNTP survey. Of the 60,031 eligible individuals, 50,309 (83.8%) participated in the survey and 50,194 (99.8%) were interviewed. Of the interviewed participants, 8744 with missing data on potential confounders were excluded in present study. The final study population for the present study consisted of 41,450 participants (69.0% of eligible individuals).

### Questionnaire

All households in the selected clusters were visited by a survey census team. During the visit, the census team observed the indoor environment of the houses and interviewed residents about environmental factors, such as the type of housing (gher, wooden house, apartment, or other), type of heating (central heating system, furnace with solid fuels, electricity, or stove with solid fuels), type of fuel used for cooking, size of gher, and average monthly household income.

All participants were also invited to a data collection site (survey venue), where they were interviewed using a structured questionnaire, which solicited information regarding demographic and socioeconomic characteristics, TB-related symptoms, number of household members, indoor smoking, previous TB history, history of contact with an active TB case, and unhealthy habits (e.g., smoking and alcohol intake). Trained health care workers measured blood pressure, height, and body weight, with participants in light clothes without shoes. Body mass index (BMI) was calculated by dividing weight by height squared (kg/m^2^). All participants underwent a chest X-ray examination using direct digital radiography. All chest X-ray images taken at the collection site were interpreted by a single experienced radiologist.

### TB diagnosis

Analysis of TB was based on survey case definitions according to the national TB guidelines and WHO recommendations. TB categories included smear positive TB and bacteriologically confirmed TB. Bacteriologically confirmed TB includes smear positive, smear negative but culture positive TB, and TB confirmed by a rapid diagnostic method such as the GeneXpert MTB/RIF assay. Presumptive TB was defined as a participant who had a cough for 2 weeks or longer at the time of the interview and/or any abnormality in the lung field or mediastinum detected by chest X-ray. These participants were asked to submit two sputum samples (one on the spot and one early the next morning) for laboratory confirmation by smear microscopy, the GeneXpert MTB/RIF assay, and liquid and solid cultures.

### Statistical analysis

All data were anonymized and entered into an electronic database for cleaning and analysis. Bacteriologically confirmed TB, non-TB, and smear positive TB groups, and solid fuel and clean fuel users, were compared using the chi-square test for categorical variables or the unpaired t-test for continuous variables. Logistic regression models were used to identify risk factors associated with TB. To incorporate nominal independent variables into the regression model, they were transformed into dichotomous variables as follows: type of fuel used for heating was grouped into solid fuels (heating with stove or furnace) and clean fuels (municipal or electric system); marital status into single (divorced or widowed) and other (married or never married); education level into lower education (none, primary, or incomplete secondary) and other (completed secondary, technical, or higher); employment into employer, self-business owner, and other (salaried employee, member of cooperative, or unpaid participant in household enterprise); smoking status into daily smoker and other (none, quit, or occasional); alcohol consumption into yes (2–4 times a month, 2–3 times a week, or at least 4 times a week) and no (none or once a month or less); and BMI into underweight (≤ 18.5 kg/m^2^) and other (> 18.5 kg/m^2^). The following variables were considered potential confounders: age, gender, education level, marital status, employment, smoking, alcohol consumption, contact with an active TB case, previous history of TB, and BMI. Given the lack of interaction effects between solid fuel use and smoking, logistic regression analysis adjusting for potential confounders was used to measure the effects of smoking, solid fuel use, and both smoking and solid fuel use on TB. Odds ratios (ORs), 95% confidence intervals (CIs), and *p* values were calculated. Statistical significance was defined as *p*<0.05 for all tests. All statistical analyses were conducted with SPSS Statistics Desktop for Japan, Version 26 (IBM Japan, Ltd., Tokyo, Japan).

## Results

A total of 248 TB cases were identified in the MNTP survey. Of these, 213 TB cases, including 75 smear positive TB cases, were analyzed in our final study sample. Mean number of years living at the same residential address was 14.8 years. General characteristics by group (bacteriologically confirmed TB, smear positive TB, and non-TB) are shown in Table 1. Significant differences were observed in gender, marital status, and education levels between bacteriologically confirmed TB and non-TB groups. Gender and education level significantly differed between non-TB and smear positive TB groups.

**Table 1:** General characteristics among study population

	Total	Non-TB	Bact TB	*P* value^a^	Smear + TB	*P* value^a^
Gender; *n* (%)						
Male	16,902 (40.8)	16,760 (40.6)	142 (66.7)	< 0.01	61 (81.3)	< 0.01
Female	24,548 (59.2)	24,477 (59.4)	71 (33.3)		14 (18.7)	
Age group; *n* (%)						
15–24	7236 (17.5)	7202 (17.5)	34 (16.0)	0.50	8 (10.7)	0.53
25–34	9413 (22.7)	9362 (22.7)	51 (23.9)		23 (30.7)	
35–44	8538 (20.6)	8501 (20.6)	37 (17.4)		15 (20.0)	
45–54	8018 (19.3)	7978 (19.3)	40 (18.8)		14 (18.7)	
55–64	4998 (12.1)	4970 (12.1)	28 (13.1)		9 (12.0)	
65+	3247 (7.8)	3224 (7.8)	23 (10.8)		6 (8.0)	
Marital status; *n* (%)						
Married	29,456 (71.1)	29,324 (71.1)	132 (62.0)	< 0.01	49 (65.3)	0.32
Never married	8593 (20.7)	8545 (20.7)	48 (22.5)		16 (21.3)	
Divorced	738 (1.8)	726 (1.8)	12 (5.6)		3 (4.0)	
Widowed	2663 (6.4)	2642 (6.4)	21 (9.9)		7 (9.3)	
Education; *n* (%)						
None	882 (2.1)	875 (2.1)	7 (3.3)	< 0.01	2 (2.7)	0.01
Primary	2242 (5.4)	2226 (5.4)	16 (7.5)		7 (9.3)	
Incompleted secondary	7289 (17.6)	7241 (17.6)	48 (22.5)		15 (20.0)	
Completed secondary	16,401 (39.6)	16,305 (39.5)	96 (45.1)		40 (53.3)	
Technical training	3636 (8.8)	3618 (8.8)	18 (8.5)		4 (5.3)	
Higher	11,000 (26.5)	10,972 (26.6)	28 (13.1)		7 (9.3)	
Employed; *n* (%)						
Unemployed	21,168 (51.1)	21,049 (51.0)	119 (55.9)	0.20	42 (56.0)	0.39
Employed	20,282 (48.9)	20,188 (49.0)	94 (44.1)		33 (44.0)	
Reason unemployed; *n* (%)						
Secondary school student	1790 (8.5)	1785 (8.5)	5 (4.2)	0.05	1 (2.4)	0.09
University/college student	3133 (14.9)	3120 (14.9)	13 (10.9)		3 (7.1)	
Retired	6864 (32.6)	6826 (32.6)	38 (31.9)		13 (31.0)	
Disabled	1634 (7.8)	1620 (7.7)	14 (11.8)		7 (16.7)	
Housewife	2996 (14.2)	2982 (14.2)	14 (11.8)		5 (11.9)	
Cannot find job	2764 (13.1)	2741 (13.1)	23 (19.3)		9 (21.4)	
Other	1873 (8.9)	1861 (8.9)	12 (10.1)		4 (9.5)	
Employment; *n* (%)						
Salaried employee	11,972 (59.1)	11,932 (59.2)	40 (42.6)	0.01	12 (36.4)	0.13
Employer	923 (4.6)	914 (4.5)	9 (9.6)		2 (6.1)	
Private business owner	6196 (30.6)	6156 (30.5)	40 (42.6)		18 (54.5)	
Member of cooperative	117 (0.6)	117 (0.6)	0 (0.0)		0 (0.0)	
Unpaid participant in household enterprise	382 (1.9)	381 (1.9)	1 (1.1)		0 (0.0)	
Other	668 (3.3)	664 (3.3)	4 (4.3)		1 (3.0)	
Residence area; *n* (%)						
Urban	23,283 (56.2)	23,159 (56.2)	124 (58.2)	0.50	41 (54.7)	0.79
Rural	18,167 (43.8)	18,078 (43.8)	89 (41.8)		34 (45.3)	
Number of years living at same address; mean (SD)						
	14.8 (14.1)	14.8 (14.1)	14.9 (13.0)	0.30	15.2 (13.9)	0.80

*TB* tuberculosis, *Bact* bacteriologically confirmed, + positive, *SD* standard deviation

Bact TB includes smear-positive TB and culture-positive or TB approved by rapid diagnostic such as Gen Xpert/RIF

Values represent mean (SD) or *N* (%)

^a^*P* values were calculated using the chi-square test for categorical variables or the unpaired *t* test for continuous variables in order to compare differences between non-TB and Bact TB groups and between non-TB and smear + TB groups

Household environmental characteristics in bacteriologically confirmed TB, non-TB, and smear positive TB groups are shown in Table 2. A majority of participants lived in a gher (32.6%), or a simple house made using wood or bricks (39.4%). The gher and simple house are not connected to a centralized heating infrastructure. Both use a simple stove or furnace to burn wood or coal for heating in the winter. With the exception of exposure to solid fuels for heating purposes, there were no significant differences in environmental factors between TB and non-TB groups, including housing type, passive tobacco smoking, presence of a separate kitchen, and average monthly household income. The distribution of households with a separate kitchen significantly differed between non-TB and smear positive TB groups. The prevalence of smear positive and bacteriologically confirmed TB cases was significantly higher in households with indoor exposure to solid fuels for heating compared to households using clean energy. Participants who smoke tobacco, drink alcohol more than twice per month, had contact with an active TB case, are underweight, and were previously diagnosed with TB were significantly more likely to have TB.

**Table 2:** Household environmental and individual factors among study population

Total		Non-TB	Bact TB	*P* value	Smear + TB	*P* value
House type; *n* (%)						
Gher	13,531 (32.6)	13,454 (32.6)	77 (36.2)	0.35	29 (38.7)	0.14
Wooden house	16,331 (39.4)	16,243 (39.4)	88 (41.3)		34 (45.3)	
Apartment	10,298 (24.9)	10,255 (24.9)	43 (20.2)		11 (14.7)	
Other	1290 (3.1)	1285 (3.1)	5 (2.3)		1 (1.3)	
Separate kitchen; *n* (%)						
Yes	21,411 (51.7)	21,313 (51.7)	98 (46.0)	0.90	30 (40.0)	0.04
No	20,039 (48.3)	19,924 (48.3)	115 (54.0)		45 (60.0)	
Exposure to solid fuel for heating; *n* (%)						
Clean	12,069 (29.1)	12,023 (29.2)	46 (21.6)	0.02	12 (16.0)	0.01
Solid fuel	29,381 (70.9)	29,214 (70.8)	167 (78.4)		63 (84.0)	
Number of family members; *n* (%)						
≤ 4	25,924 (62.5)	25,784 (62.5)	140 (65.7)	0.30	47 (62.7)	0.98
> 5	15,526 (37.5)	15,453 (37.5)	73 (34.3)		28 (37.3)	
Exposure to tobacco smoke inside home, *n*						
Never	17,264 (41.7)	17,181 (41.7)	83 (39.0)	0.70	28 (37.3)	0.42
Occasional	10,554 (25.5)	10,499 (25.5)	55 (25.8)		17 (22.7)	
Daily	13,632 (32.9)	13,557 (32.9)	75 (35.2)		30 (40.0)	
Household monthly income (₮)^a^; *n* (%)						
≤ 500,000	22,155 (53.4)	22,038 (53.4)	117 (54.9)	0.30	46 (61.3)	0.38
500,001–1,000,000	14,948 (36.1)	14,871 (36.1)	77 (36.2)		25 (33.3)	
1,000,001–1,500,000	2719 (6.6)	2711 (6.6)	8 (3.8)		2 (2.7)	
≥ 1,500,001	1628 (3.9)	1617 (3.9)	11 (5.2)		2 (2.7)	
Smoking; *n* (%)						
Never	29,781 (71.9)	29,683 (72.0)	98 (46.0)	< 0.01	20 (26.7)	< 0.01
Quit	1259 (3.0)	1248 (3.0)	11 (5.2)		7 (9.3)	
Occasional	1396 (3.4)	1387 (3.4)	9 (4.2)		5 (6.7)	
Daily	9014 (21.7)	8919 (21.6)	95 (44.6)		43 (57.3)	
Alcohol consumption; *n* (%)						
Never	22,351 (53.9)	22,266 (54.0)	85 (39.9)	< 0.01	27 (36.0)	< 0.01
Once a month or less	15,892 (38.3)	15,801 (38.3)	91 (42.7)		30 (40.0)	
2-4 times a month	2837 (6.8)	2812 (6.8)	25 (11.7)		12 (16.0)	
2–3 times a week	265 (0.6)	257 (0.6)	8 (3.8)		4 (5.3)	
At least 4 times a week	105 (0.3)	101 (0.2)	4 (1.9)		2 (2.7)	
Diabetes; *n* (%)						
No	17,801 (43.0)	17,717 (43.0)	84 (39.4)	0.09	32 (42.7)	0.67
Yes	1011 (2.4)	1002 (2.4)	9 (4.2)		3 (4.0)	
Unknown	22,638 (54.6)	22,518 (54.6)	120 (56.3)		40 (53.3)	
Contact with active TB; *n* (%)						
No	35,020 (84.5)	34,862 (84.5)	158 (74.2)	< 0.01	54 (72.0)	0.01
Yes	6430 (15.5)	6375 (15.5)	55 (25.8)		21 (28.0)	
BMI; *n* (%)						
Normal	18,779 (45.3)	18,627 (45.2)	152 (71.4)	< 0.01	54 (72.0)	< 0.01
Overweight	13,206 (31.9)	13,176 (32.0)	30 (14.1)		7 (9.3)	
Obese class I	6096 (14.7)	6089 (14.8)	7 (3.3)		1 (1.3)	
Obese class II	1543 (3.7)	1543 (3.7)	0 (0.0)		0 (0.0)	
Obese class III	470 (1.1)	469 (1.1)	1 (0.5)		0 (0.0)	
Underweight	1356 (3.3)	1333 (3.2)	23 (10.8)		13 (17.3)	
Previous history of TB; *n* (%)						
No	39,785 (96.0)	39,612 (96.1)	173 (81.2)	< 0.01	52 (69.3)	< 0.01
Yes	1665 (4.0)	1625 (3.9)	40 (18.8)		23 (30.7)	

*TB* tuberculosis, *Bact* bacteriologically confirmed, *₮* tugrik

Bact TB includes smear-positive TB and culture-positive or TB approved by rapid diagnostic method such as Gen Xpert/RIF

*P* values were calculated using the chi-square test to compare differences between non-TB and Bact TB groups and between non-TB and smear+ TB groups

^a^Average monthly household income based on tugrik (₮) Mongolian currency $1 = 1800 ₮ in 2015

Table 3 compares the general characteristics of participants who use solid fuels and clean fuels. Participants who were aged > 25 years, who were married, who had a low level of education, who were unemployed, and who were rural residents were more likely to use solid fuels for heating than clean fuels. No significant gender difference was observed between solid fuel and clean fuel users. Household environment and individual factors significantly differed between solid fuel and clean fuel users (Table 4). Families with a lower income were more likely to use solid fuels, and families that used solid fuels were more exposed to tobacco smoke inside the home than families that used clean fuels.

**Table 3:** General characteristics of clean fuel users and solid fuel user

	Total	Clean fuel user	Solid fuel user	*P* value
Gender; *n* (%)				
Female	24,818 (59.9)	7277 (60.3)	17,541 (59.7)	0.30
Male	16632 (40.1)	4792 (39.7)	11,840 (40.3)	
Age group; *n* (%)				
15–24	7236 (17.5)	2678 (22.2)	4558 (15.5)	< 0.01
25–34	9413 (22.7)	2660 (22.0)	6753 (23.0)	
35–44	8538 (20.6)	2320 (19.2)	6218 (21.2)	
45–54	8018 (19.3)	2106 (17.4)	5912 (20.1)	
55–64	4998 (12.1)	1360 (11.3)	3638 (12.4)	
65+	3247 (7.8)	945 (7.8)	2302 (7.8)	
Marital status; *n* (%)				
Married	29,456 (71.1)	7918 (65.6)	21,538 (73.3)	< 0.01
Never married	8593 (20.7)	3093 (25.6)	5500 (18.7)	
Divorced	738 (1.8)	264 (2.2)	474 (1.6)	
Widowed	2663 (6.4)	794 (6.6)	1869 (6.4)	
Education; *n* (%)				
None	882 (2.1)	108 (0.9)	774 (2.6)	< 0.01
Primary	2242 (5.4)	207 (1.7)	2035 (6.9)	
Incomplete secondary	7289 (17.6)	1146 (9.5)	6143 (20.9)	
Completed secondary	16,401 (39.6)	4244 (35.2)	12,157 (41.4)	
Technical training	3636 (8.8)	1027 (8.5)	2609 (8.9)	
Higher	11,000 (26.5)	5337 (44.2)	5663 (19.3)	
Employed; *n* (%)				
Unemployed	21,168 (51.1)	6013 (49.8)	15,155 (51.6)	< 0.01
Employed	20,282 (48.9)	6056 (50.2)	14,226 (48.4)	
Reason unemployed; n (%)				
Secondary school student	1790 (8.5)	456 (7.6)	1334 (8.9)	< 0.01
University / college student	3133 (14.9)	1657 (27.6)	1476 (9.8)	
Retired	6864 (32.6)	1899 (31.7)	4965 (33.0)	
Disabled	1634 (7.8)	329 (5.5)	1305 (8.7)	
Housewife	2996 (14.2)	740 (12.3)	2256 (15.0)	
Cannot find job	2764 (13.1)	344 (5.7)	2420 (16.1)	
Other	1873 (8.9)	572 (9.5)	1301 (8.6)	
Employment; n (%)				
Salaried employee	11,972 (59.1)	4090 (67.7)	7882 (55.4)	< 0.01
Employer	923 (4.6)	326 (5.4)	597 (4.2)	
Private business owner	6196 (30.6)	1565 (25.9)	4631 (32.6)	
Member of cooperative	117 (0.6)	11 (0.2)	106 (0.7)	
Unpaid participant in household enterprise	382 (1.9)	15 (0.2)	367 (2.6)	
Other	668 (3.3)	36 (0.6)	632 (4.4)	
Residence area; *n* (%)				
Urban	23,283 (56.2)	10,550 (87.4)	12,733 (43.3)	< 0.01
Rural	18,167 (43.8)	1519 (12.6)	16,648 (56.7)	

Values represent *N* (%)

*P* values were calculated using the chi-square test for categorical variables in order to compare differences between clean fuel user and solid fuel user

**Table 4:** Household environmental and individual factors among solid fuel user and clean fuel user

	Total	Clean fuel user	Solid fuel user	*P*-value
Houses type; n (%)				
Gher	13531 (32.6)	300 (2.5)	13231 (45.0)	<0.01
Wooden house	16331 (39.4)	786 (6.5)	15545 (52.9)	
Apartment	10298 (24.8)	9989 (82.8)	309 (1.1)	
Other	1290 (3.1)	994 (8.2)	296 (1.0)	
Separate kitchen; n (%)				
Yes	21411 (51.7)	8982 (74.4)	12429 (42.3)	<0.01
No	20039 (48.3)	3087 (25.6)	16952 (57.7)	
Number of family members; n (%)				
≤ 4	25924 (62.5)	7797 (64.6)	18127 (61.7)	<0.01
> 5	15526 (37.5)	4272 (35.4)	11254 (38.3)	
Exposure to tobacco smoke inside home; n (%)				
Never	17264 (41.7)	5231 (43.3)	12033 (41.0)	<0.01
Occasional	10554 (25.5)	3499 (29.0)	7055 (24.0)	
Daily	13632 (32.9)	3339 (27.7)	10293 (35.0)	
Household monthly income (₮); n (%)				
≤ 500,000	22155 (53.4)	3828 (31.7)	18327 (62.4)	<0.01
500,001-1,000,000	14948 (36.1)	5550 (46.0)	9398 (32.0)	
1,000,001-1,500,000	2719 (6.6)	1576 (13.1)	1143 (3.9)	
≥ 1,500,001	1628 (3.9)	1115 (9.2)	513 (1.7)	
Smoking; n (%)				
Never	29781 (71.8)	8756 (72.5)	21025 (71.6)	<0.01
Quit	1259 (3.0)	380 (3.1)	879 (3.0)	
Occasional	1396 (3.4)	504 (4.2)	892 (3.0)	
Daily	9014 (21.7)	2429 (20.1)	6585 (22.4)	
Alcohol consumption; n (%)				
Never	22351 (53.9)	5860 (48.6)	16491 (56.1)	<0.01
Once a month or less	15892 (38.3)	5182 (42.9)	10710 (36.5)	
2-4 times a month	2837 (6.8)	933 (7.7)	1904 (6.5)	
2-3 times a week	265 (0.6)	69 (0.6)	196 (0.7)	
At least 4 times a week	105 (0.3)	25 (0.2)	80 (0.3)	
Diabetes; n (%)				
No	17801 (42.9)	1484 (12.3)	16317 (55.5)	<0.01
Yes	1011 (2.4)	387 (3.2)	624 (2.1)	
Unknown	22638 (54.6)	10198 (84.5)	12440 (42.3)	
Contact with active TB; n (%)				
No	35020 (84.5)	9934 (82.3)	25086 (85.4)	<0.01
Yes	6430 (15.5)	2135 (17.7)	4295 (14.6)	
BMI; n (%)				
Normal	18779 (45.3)	5286 (43.8)	13493 (45.9)	<0.01
Overweight	13206 (31.9)	3879 (32.1)	9327 (31.7)	
Obese class I	6096 (14.7)	1857 (15.4)	4239 (14.4)	
Obese class II	1543 (3.7)	484 (4.0)	1059 (3.6)	
Obese class III	470 (1.1)	127 (1.1)	343 (1.2)	
Underweight	1356 (3.3)	436 (3.6)	920 (3.1)	
Previous history of TB; n (%)				
No	39785 (96.0)	11558 (95.8)	28227 (96.1)	0.10
Yes	1665 (4.0)	511 (4.2)	1154 (3.9)	

*TB* tuberculosis, *BMI* body mass index

*P* values were calculated using chi-square test to compare differences between clean fuel user and solid fuel user

^a^Average monthly household income based on tugrik (₮) Mongolian currency $1 = 1800 ₮ in 2015

**Fig. 3:**
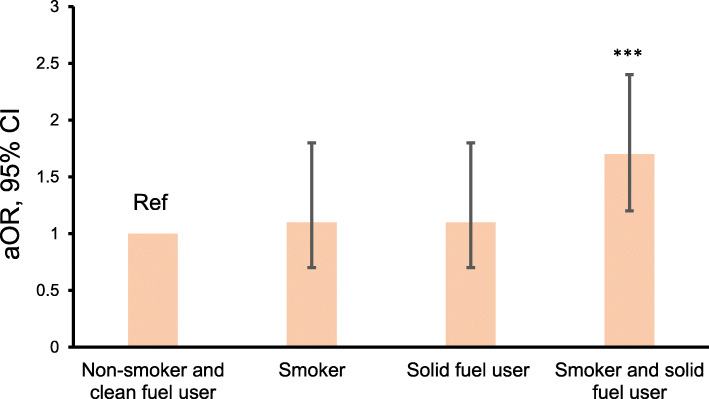
Combined effect of smoking and exposure to solid fuels on TB. Adjusted odds ratios were estimated by logistic regression after adjusting for age, gender, education level, marital status, employment, alcohol consumption, contact with an active TB case, underweight status, and previous history of tuberculosis. *P* < 0.05 was considered statistically significant. ***P < 0.01, compared with non-smoking clean fuel user. *aOR* adjusted odds ratio, *CI* confidence interval, % percentage

Table 5 shows factors associated with bacteriologically confirmed TB and smear positive TB. Male gender, having a lower education level than secondary education, being divorced or widowed, being an employer or a private business owner, being a daily smoker, drinking alcohol more than twice a month, having contact with an active TB case, being underweight, being exposed to solid fuels for heating, and having a history of TB were significantly related to TB by univariate logistic regression analysis. In the multivariate logistic regression analysis, which included these factors plus age in the same model, exposure to solid fuels for heating was significantly associated with bacteriologically confirmed TB (OR: 1.5; 95% CI: 1.1–2.1; *p* = 0.02) and smear positive TB (OR = 2.1; 95% CO: 1.1–4.0; *p* = 0.01). Figure 3 shows the adjusted ORs (aORs) of smoking, exposure to solid fuels for heating, or exposure to both for bacteriologically confirmed TB. Both exposure to smoke from tobacco and solid fuels for heating were significantly associated with bacteriologically confirmed TB after adjusting for age, gender, marital status, education level, employment, being underweight, alcohol consumption, contact with an active TB case, and previous history of TB.

**Table 5:** The factors associated with bacteriologically confirmed TB and smear positive TB among study population

Independent variables	Prevalence of Bact TB (%)	Univariate analysis		Multivariate analysis			
		Bact TB		Bact TB		Smear + TB	
		cOR (95%, CI)	*P* value	aOR (95%, CI)	*P* value^a^	aOR (95%, CI)	*P* value^a^
Gender							
Male	0.8	2.9 (2.2–3.9)	0.01	2.2 (1.6–3.1)	< 0.01	4.2 (2.3–8.2)	< 0.01
Female	0.3	1.0	–	1.0	–	1.0	–
Exposure to solid fuel for heating							
Yes	0.6	1.5 (1.1–2.1)	0.01	1.5 (1.1–2.1)	0.02	2.1 (1.1–4.0)	0.01
No	0.4	1.0	–	1.0	–	1.0	–
Education							
Lower than incomplete secondary	0.7	1.5 (1.1–2.0)	0.01	1.1 (0.9–1.6)	0.35	1.0 (0.6–1.7)	0.98
Higher than complete secondary	0.5	1.0	–	1.0	–	1.0	–
Marital status							
Divorced or widow	1.0	2.1 (1.4–3.0)	0.01	2.5 (1.6–3.8)	0.01	2.7 (1.3–5.6)	0.01
Married or never married	0.5	1.0	–	1.0	–	1.0	–
Employment							
Employer and self-business owner	0.7	1.4 (1.1–2.0)	0.03	1.4 (0.9–1.9)	0.06	1.4 (0.8–2.4)	0.23
Others	0.5	1.0	–	1.0	–	1.0	–
Smoking							
Daily	1.1	2.9 (2.2–3.8)	0.01	1.8 (1.3–2.5)	< 0.01	2.1 (1.2–3.5)	0.01
Never, quit, occasional	0.4	1.0	–	1.0	–	1.0	–
Alcohol intake							
Yes	1.2	2.5 (1.8–3.6)	< 0.01	1.4 (0.9–2.1)	0.07	1.5 (0.9–2.8)	0.13
No	0.5	1.0	–	1.0	–	1.0	–
Contacts with active TB							
Yes	0.9	1.9 (1.4–2.6)	< 0.01	1.7 (1.2–2.3)	0.01	1.6 (0.9–2.7)	0.13
No	0.5	1.0	–	1.0	–	1.0	–
Underweight							
BMI ≤ 18.5 kg/m^2^	1.7	3.6 (2.3–5.6)	< 0.01	3.6 (2.3–5.7)	< 0.01	7.1 (3.7–13.5)	< 0.01
BMI > 18.5 kg/m^2^	0.5	1.0	–	1.0	–	1.0	–
History of TB							
Yes	2.4	5.6 (4.0–8.0)	< 0.01	4.3 (3.0–6.2)	< 0.01	7.5 (4.4–12.6)	< 0.01
No	0.4	1.0	–	1.0	–	1.0	–

*TB* tuberculosis, *Bact* bacteriologically confirmed, + positive, *cOR* crude odds ratio, *aOR* adjusted odds ratio, *CI* confidence interval, *BMI* body mass index

Smoking: yes: tobacco smoking daily; alcohol consumption: yes: more than 2–4 times per month

BMI: underweight: ≤ 18.5 kg/m^2^. Adjusted effects of the independent variables on TB in the study population

Both crude and adjusted odds ratio were estimated by logistic regression. Unadjusted odds ratios were based on separate logistic regression for independent variables

TB case, underweight status, previous history of tuberculosis and exposure to solid fuel for heating

^a^All independent variables were included in the same model: age, gender, education level, marital status, employment, smoking, alcohol intake, contact with active TB case, underweight status, previous history of tuberculosis and exposure to solid fuel for heating

## Discussion

The present large-scale study of a representative Mongolian adult population found a significant positive association between exposure to solid fuels for heating and TB. This association was independent of potential confounding factors, such as gender, smoking, marital status, BMI, contact with an active TB case, and previous history of TB. IAP from household solid fuel combustion may be a risk factor for TB in the Mongolian population, which spends most of the time at home indoors due to the cold climate.

In 2017, household IAP contributed to 1.8 million global deaths and 60.9 million disability adjusted life years (DALYs), and infectious respiratory diseases including TB accounted for most of the respiratory burden, with 27.4 million DALYs [7]. Although TB and IAP are both pressing public health issues in Mongolia, the present study is the first to report an association between IAP due to solid fuel combustion and TB in Mongolia. Due to extreme cold and long heating season, it is common for households to keep their doors and windows closed, which reduces the air circulation indoors, concentration of the pollutants released from burning solid fuels increases the exposure to respirable pollutants on individual level. Prolonged exposure to such pollutants impairs the normal clearance of secretions on the tracheobronchial mucosal surface and thus may allow a causative organism mycobacterium TB, to escape the first level of host defenses which prevent bacilli from reaching the alveoli [5].

Given that people spend 90% of their lifetime in indoor settings, indoor air quality is a major risk factor for human health [24, 25]. Some epidemiological studies have reported an association between solid fuel smoke and TB. In a case-control study conducted in Mexico, household IAP exposure was found to facilitate the development of active TB, and exposure to smoke from biomass fuels for more than 20 years led to a 3-fold higher incidence of active TB than controls (OR: 3.3; 95% CI: 1.06–10.30) [26]. A hospital-based case-control study by Pokhrel et al. found that exposure to IAP was 3.4 times more common in TB cases than in controls [27]. A meta-analysis, which included a systematic review of 12 papers, reported a 30% higher risk of developing TB in individuals exposed to IAP (OR: 1.30; 95% CI: 1.04–1.62; *p* < 0.02) [19]. Another meta-analysis concluded that the risk of active TB depends on the type of fuel used, with the highest risk (43% increased risk) being associated with burning solid fuels [25]. A recent meta-analysis reported that IAP is associated with the risk of contracting TB (relative risk: 1.68; 95%, CI: 1.108–2.542; *p* < 0.014) [28].

The combustion of solid fuels emits many chemicals which impact human health, including PM, carbon dioxide, carbon monoxide, sulfur dioxide (SO_2_), sulfur trioxide, nitrogen dioxide, and nitric oxide [29]. There is increasing evidence that PM exposure weakens anti-mycobacterial host immunity [30, 31]. Chronic PM exposure accompanied by high constitutive expression of pro-inflammatory cytokines results in relative cellular unresponsiveness [31, 32]. Eighty percent of the total global exposure to airborne PM occurs indoors in developing countries [33]. PM_2.5_ has been reported to affect lung pathology, with smear positive TB patients being more exposed to PM_2.5_ than smear negative TB patients [34]. Moreover, chronic exposure to PM_10_ ≥ 50 μg/m^3^ was associated with an increase in the time required for TB positive sputum culture conversion [35]. In the present study, people exposed to IAP from household solid fuel use were more likely to have smear positive TB than bacteriologically confirmed TB, and exposure to smoke from tobacco were also associated with bacteriologically confirmed TB. The indoor PM_2.5_ concentration is very high in ghers and houses with stoves using semi-coke coal, with estimates of 107.0 μg/m^3^ in winter months average, which is higher than the permissible concentration in the WHO air quality guidelines (i.e., not exceed 10 μg/m^3^ annual mean or 25 μg/m^3^ 24-h mean) [36]. SO_2_ is also a major pollutant from solid fuel combustion, and SO_2_ from coal burning is associated with persistent cough symptoms among schoolchildren in urban and suburban Mongolia [37].

In Mongolia, 45.2% of households live in traditional ghers and 29.5% live in ordinary wooden houses [8]. Over 95% of households living in ghers use solid fuels including coal for everyday cooking and heating [38]. The traditional gher is a portable circular wood framed dwelling covered in multiple layers of wool felt. Heating is provided by a stove located at the center of the gher, and a chimney directs the fuel smoke through the central roof vent. In Mongolia, TB cases show seasonality, sharply rising in the spring from March to May. UB is the coldest capital city in the world, with temperatures reaching minus 40 °C during the night in winter. People spend most of their time indoors, and thus transmissibility of TB increases, as people are exposed to solid fuel smoke at home [39, 40].

We also found that TB is more common among males than females, and that tobacco smoking is associated with TB. Compared to non-smokers, smokers have an increased risk of developing TB (OR: 1.8; 95% CI: 1.3–2.5; *p* < 0.01). This finding is consistent with previous studies. In addition, there was positive association between tobacco smoking and solid fuel use in the present study. Therefore, tobacco smoking may be one of potential confounding factors. According to a WHO report, the global TB incidence is higher in males than in females, with male-to-female ratios of TB ranging from 1.3 in the Eastern Mediterranean to 2.1 in the Western Pacific region [3]. This gender difference in incidence might be explained by the higher rate of smoking among Mongolian men than women (males, 46.3%; females, 6.8%) [41]. In the present study, smoking was more prevalent in males compared to females (males, 44%; females, 6%). Plenty of epidemiological and biological studies provide insight into the biological mechanism underlying the association between tobacco smoking and TB. Tobacco smoke exposure attenuates cytokine production and TB killing by macrophages, and exposure to nicotine impairs the anti-TB defense of macrophages by two mechanisms, including the inhibition of autophagy and activation of immunosuppressive Treg cells [42].

Numerous epidemiological studies demonstrate that both the exposure from active and passive smoking have been shown to be associated with TB infection and the transmission from being infected to developing TB disease [43–45]. However, our present study could not find the relationship between passive tobacco exposure and TB association. Passive smoking exposure is lower than that experienced by active smokers, while the smoke is generally similar and contains the same gases and particles including a wide range of irritating compounds and carcinogens [46]. Indoor PM2.5 concentrations can become extremely high when burning solid fuels than tobacco smoking, therefore a relatively small effect size might also partly explain why it has previously proved so difficult to establish such a relationship in this study.

The present study has several strengths. First, we used data from a nationally representative population-based survey which targeted households throughout the country. Thus, our sample size was very large, reducing potential type 2 error. Second, detailed information about potential risk factors for TB were recorded, allowing us to comprehensively adjust for confounders. Third, TB was diagnosed by laboratory test results rather than subjectively by self-report or based on a clinically-diagnosed previous history of the disease.

The present study also has some limitations worth noting. First, as with other observational studies, associations observed may be due to unmeasured confounders. However, the associations between household solid fuel use and TB reported in the present study were independent of other potential confounders such as smoking, gender, marital status, education, alcohol intake, BMI, contact with an active TB case, and previous history of TB. Second, we adopted a cross-sectional design. Data on assessed variables were obtained only at the time of recruitment, and thus the duration of risk factors and its impact to the individual’s level could not be assessed. Moreover, the exposure to solid fuel smoke from cooking and heating was self-reported, and the duration of exposure to solid fuels and concentration of pollutants in indoor settings were not measured. That said, the study team visited every household and confirmed the type of dwelling and stoves used in order to minimize recall bias.

## Conclusion

This large, population-based cross-sectional analysis showed that exposure to solid fuels for heating is associated with active TB, including smear positive TB, independently of several confounding factors, in Mongolian adults. Moreover, the combination of smoking and solid fuel use for heating is associated with developing active TB. A greater awareness of and more education on the use of solid fuels is needed, given its relevance as a source of IAP and relationship with TB.

## Data Availability

The dataset generated during the current study is not publicly available, but is available from the corresponding author on reasonable request.

## Abbreviations

aOR: Adjusted odds ratio
BMI: Body mass index
DALY: Disability adjusted life years
HIV: Human immunodeficiency virus
IAP: Indoor air pollution
MNTP Survey: Mongolian National Tuberculosis Prevalence Survey
OR: Odds ratio
PM: Particulate matter
PSUs: Primary sampling units
SO_2_: Sulfur dioxide
TB: Tuberculosis
UB: Ulaanbaatar
WHO: World Health Organization
