# Correction to: Association between household solid fuel use and tuberculosis: cross-sectional data from the Mongolian National Tuberculosis Prevalence Survey

**DOI:** 10.1186/s12199-021-01006-3

**Published:** 2021-09-07

**Authors:** Munkhjargal Dorjravdan, Katsuyasu Kouda, Tsolmon Boldoo, Naranzul Dambaa, Tugsdelger Sovd, Chikako Nakama, Toshimasa Nishiyama

**Affiliations:** 1 Department of Hygiene and Public Health, Kansai Medical University, 2-5-1 Shin-machi, Hirakata, Osaka, 573-1010 Japan; 2 Tuberculosis Surveillance and Research Department, National Center for Communicable Disease, Nam Yan Ju Street, Bayanzurkh district, Ulaanbaatar, 13701 Mongolia; 3 Swiss Tropical and Public Health Institute, Socinstrasse 57, CH-4051 Basel, Switzerland

**Correction to:** ***Environ Health Prev Med*** **26, 76 (2021).**

**https://doi.org/10.1186/s12199-021-00996-4**

Following the publication of the original article [1] the authors noticed that the correction requested in Table 4 was not implemented. The “₮ “should be inserted in the “()” following “Household monthly income” as shown below.

“Household monthly income (₮); n (%)”.

The original article [1] has been updated.
