# Correction to: Figure 3 in Association between household solid fuel use and tuberculosis: cross-sectional data from the Mongolian National Tuberculosis Prevalence Survey

**DOI:** 10.1265/ehpm.22-00271

**Published:** 2022-12-21

**Authors:** Munkhjargal Dorjravdan, Katsuyasu Kouda, Tsolmon Boldoo, Naranzul Dambaa, Tugsdelger Sovd, Chikako Nakama, Toshimasa Nishiyama

**Affiliations:** 1 Department of Hygiene and Public Health, Kansai Medical University, 2-5-1 Shin-machi, Hirakata, Osaka 573-1010, Japan; 2 Tuberculosis Surveillance and Research Department, National Center for Communicable Disease, Nam Yan Ju Street, Bayanzurkh district, Ulaanbaatar 13701, Mongolia; 3 Swiss Tropical and Public Health Institute, Socinstrasse 57, CH-4051 Basel, Switzerland

**Correction to: *Environ Health Prev Med* 26, 76 (2021)**

**https//doi.org/10.1186/s12199-021-00996-4**

Following the publication of the original article [1] the authors noticed a graphing error in the Figure 3. The Figure 3 needs to be replaced with the updated graph as shown below. In addition, “*P <0.05, compared with non-smoking clean fuel user” should be inserted in front of the sentence “***P <0.01, compared with non-smoking clean fuel user” in legend of the Figure 3. Moreover, “Solid fuel user was significantly associated with bacteriologically confirmed TB independent of smoking and other confounders.” should be placed behind the sentence “Both exposure to smoke from tobacco and solid fuels for heating were significantly associated with bacteriologically confirmed TB after adjusting for age, gender, marital status, education level, employment, being underweight, alcohol consumption, contact with an active TB case, and previous history of TB.”

**Fig. 3:**
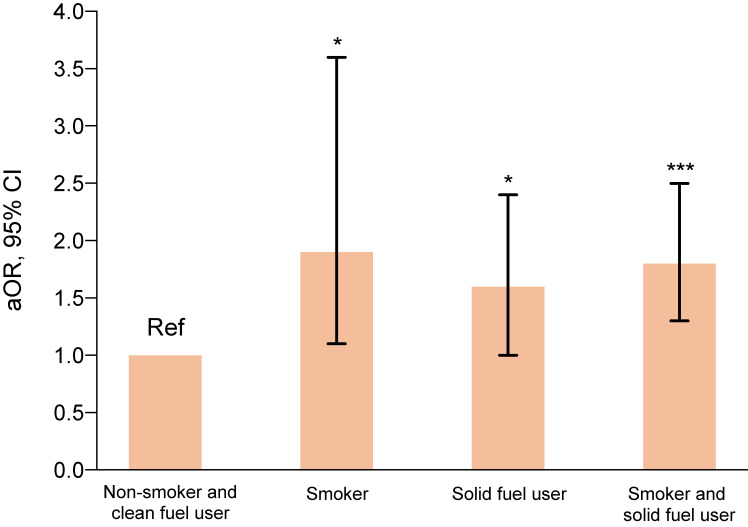


The original article [1] has been updated.
